# MRI Detection of Unknown Primary Tumours in the Head and Neck: What Is the Expected Normal Asymmetry in the Size of the Palatine Tonsils?

**DOI:** 10.3390/diagnostics15060788

**Published:** 2025-03-20

**Authors:** Kaijing Mao, Qi Yong H. Ai, Kuo Feng Hung, Irene O. L. Tse, Ho Sang Leung, Yannis Yan Liang, Yu Chen, Lun M. Wong, W. K. Jacky Lam, Ann D. King

**Affiliations:** 1 Department of Health Technology and Informatics, The Hong Kong Polytechnic University, Hung Hom, Kowloon, Hong Kong SAR, China; kjmao@connect.hku.hk (K.M.); liangyan@link.cuhk.edu.hk (Y.Y.L.); 2 Restorative Dental Sciences, Faculty of Dentistry, The University of Hong Kong, Hong Kong SAR, China; 3 Department of Imaging and Interventional Radiology, The Chinese University of Hong Kong, Shatin, New Territories, Hong Kong SAR, China; lhs655@ha.org.hk (H.S.L.); lun.m.wong@cuhk.edu.hk (L.M.W.); 4 Department of Diagnostic Radiology, The University of Hong Kong, Pokfulam, Hong Kong SAR, China; 5 Applied Oral Sciences & Community Dental Care, Faculty of Dentistry, The University of Hong Kong, Hong Kong SAR, China; hungkfg@hku.hk; 6 Department of Chemical Pathology, The Chinese University of Hong Kong, Shatin, New Territories, Hong Kong SAR, China; irenetol@cuhk.edu.hk (I.O.L.T.); jwaikeilam@cuhk.edu.hk (W.K.J.L.); 7 Department of Radiology, Peking Union Medical College Hospital, Chinese Academy of Medical Sciences, Dong Cheng District, Beijing 100730, China; bjchenyu@126.com

**Keywords:** tonsillar asymmetry, MRI, head and neck cancer detection

## Abstract

**Background/Objectives:** The detection of unknown primary tumours in the palatine tonsils (PTs) on imaging relies heavily on asymmetry in size between the right and left sides, but the expected normal range in asymmetry is not well documented. This study aimed to document the expected range of asymmetry in the size of the PTs in adults without cancer. **Methods:** This retrospective study evaluated 250 pairs of normal PTs on MRIs of adults without head and neck cancer. The size (volume, V) of the PTs on the left and right sides were measured, and the percentage difference in volume (ΔV%) between the two sides was calculated. An additional analysis of PT volumes in 29 patients with ipsilateral early-stage palatine tonsillar cancer (PTCs) was performed. **Results:** In patients without PTC, the normal PTs had a mean volume of 3.0 ± 1.7 cm^3^, and there was a difference in size between the left and right PTs, showing a median ΔV% of 11.6% (range: 0.1–79.0%); most patients had a ΔV% of ≤40% (95%) for PTs. In patients with ipsilateral PTC, the normal PT had a smaller size compared with PTC (*p* < 0.01), showing a median ΔV% of 132.9% (range: 8.5–863.2%). Compared with patients without PTC, those with PTC showed a greater ΔV% (*p* < 0.01). An optimal ΔV% threshold of >39.6% achieved the best accuracy of 95% for identifying PTC. **Conclusions:** PTs are asymmetrical in size in adults without PTC. An additional analysis involving patients with PTC confirmed a threshold of ΔV% of 40% for PTs, which may be clinically valuable to help detect pathology using MRI.

## 1. Introduction

Head and neck squamous cell carcinoma (SCC) is a common cancer in the aerodigestive tract, with a high propensity to spread to the regional lymph nodes, which may be a presenting symptom of the disease [1,2,3]. Primary tumour site detection is crucial for treatment planning for patients presenting with metastatic neck nodes. In most patients, the primary site is easily identified by clinical and endoscopic examinations [4,5,6]. However, 5–10% of patients may have a primary tumour that is too small to be visible, and further investigation for patients with an unknown primary tumour requires imaging with positron emission tomography–computed tomography (PET/CT) and magnetic resonance imaging (MRI) [7,8,9].

The lymphoid tissues of the palatine tonsils (PTs) are common sites for hidden tumours [10,11], but small tumours may be obscured on the PET imaging by the normal increased 18F-fluorodeoxyglucose (FDG) uptake at these sites. Therefore, cancer detection often relies on asymmetry in size between the right and left sides of the oropharynx on the anatomical images from CT or MRI [10,12,13]. However, some degree of asymmetry in size is often observed in patients without cancer who have either normal tonsils or tonsils that are enlarged by benign hyperplasia or inflammation [14,15]. Furthermore, we have observed that patients with prominent lymphoid tissues in the palatine tonsils often have prominent benignly appearing reactive neck nodes.

Therefore, this study aimed to establish a normal quantitative reference range for asymmetry in the palatine tonsils in adults without head and neck cancer. The sizes were documented and compared using the volume of the left and right PTs on MRI. We also compared differences in size between normal PTs and early-stage palatine tonsillar cancer (PTC), as well as differences in the size asymmetry of the bilateral palatine tonsils in patients without PTC and patients with ipsilateral PTC. We also investigated potential associations between the size of tonsils and lymphatic tissues in the head and neck by correlating the volume of tonsils with age, cigarette consumption, and the size of the ipsilateral nodes in the upper neck.

## 2. Methods and Materials

### 2.1. Patients

The local institutional ethics review board approved this retrospective study. Given the study’s retrospective nature, the requirement for written consent was waived. To build up the quantitative normal reference for the size symmetry of normal PTs, this study firstly analysed the upper head and neck MRI scans of 250 adult patients without PTC who fulfilled the following inclusion criteria: (1) patients who were initially prospectively recruited for head and neck cancer screening studies using Epstein–Barr virus (EBV) biomarkers between 2005 and 2016 and referred to our institution for head and neck MRI [16,17]; (2) MRI coverage included the whole of the palatine tonsils; (3) patients with measurable palatine tonsils (defined as a maximum axial diameter of larger than 3 mm); and (4) no previous history of head and neck cancer and no head and neck cancer diagnosed during a minimum follow-up period of 2 years. Furthermore, this study analysed the pre-treatment upper head and neck MRI scans of 29 patients with surgically proven squamous cell carcinoma in the ipsilateral PT, who fulfilled the following inclusion criteria: (1) patients who had pre-treatment MRI showing stage T1-2 PTC between 2021 and 2024; (2) MRI coverage included the whole of the palatine tonsils; and (3) patients with measurable palatine tonsils (defined as a maximum axial diameter of larger than 3 mm). Patients with a history of tonsil surgery or MR images were unable to be evaluated due to artefacts being excluded from the study. Patient demographics were also recorded for analysis.

### 2.2. MRI Acquisition and Analysis

The upper head and neck MRI was performed using a 1.5 T or 3 T whole-body MRI system (Philips Healthcare, Best, The Netherlands). The analysis was performed on the axial fat-suppressed T2-weighted images (repetition time/echo time of 2500–4000/80–100 ms; field of view of 22 cm; slice thickness of 4 mm) for the PTs.

**Figure 1:**
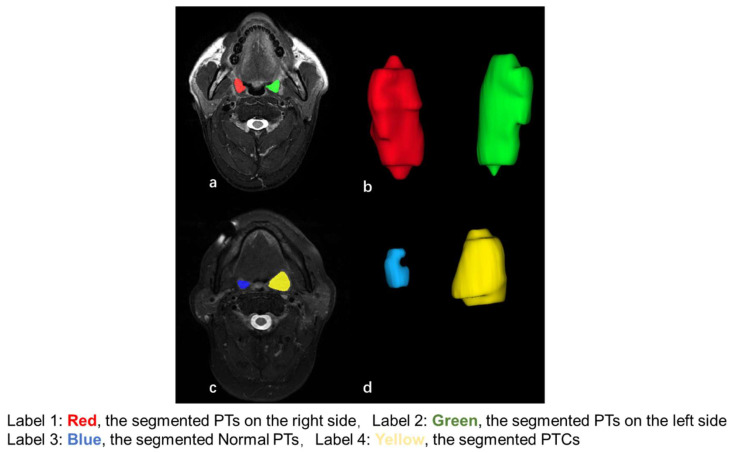
The axial T2-weighted fat-suppressed MR images and reconstructed three-dimensional images of bilateral palatine tonsils (PTs) of a patient without palatine tonsillar cancer (PTC) (**a**,**b**) and a patient with stage T1 PTC (**c**,**d**). The patient without PTC showed similar sizes in the left (green label) and right (red label) PTs, with a percentage difference in volume of 8.4%. The patient with ipsilateral PTC showed an enlarged PTC on the left side (yellow label) and an atrophic normal PT on the right side (blue label), with a percentage difference in volume of 863.2%.

The solid components of PTs on the left and right sides in patients without and with PTC were manually segmented by a researcher with 10 years of experience in head and neck MRI using the open-source software ITK-SNAP (version 3.4.0; http://www.itksnap.org) (Figure 1). Another experienced clinician with 5 years of experience in oral and maxillofacial radiology also performed segmentations for the PTs on 50 randomly selected MRIs for the inter-observer assessment. A three-dimensional size feature (volume) was calculated using the triangle mesh of the region of interest (ROI).

### 2.3. Node Size

Five cervical nodal groups in the upper neck comprising (1) retropharyngeal, (2) submandibular, (3) jugulodigastric, (4) Level IIa (other than jugulodigastric), and (5) Level IIb nodes were analysed, and the short axial diameter (SAD) of the largest node in each group on each side of the neck was measured. Only nodes with an SAD of larger than 2.0 mm were considered measurable and included in this study. The measurement was performed by the same researcher with 10 years of experience in head and neck MRI and were reported previously [18].

### 2.4. Statistical Analysis

In patients without PTC, the difference in volume (V) between the left and right PTs in patients without PTC was analysed using the paired *t*-test; the percentage difference in volume (ΔV%) using the absolute value of (right value–left value)/left value × 100%; and the mean and 95% confidence intervals (CIs) of the ΔV%. The V of the palatine tonsil on each side was correlated with the SADs of the ipsilateral upper neck nodes and age using the Pearson correlation test, and the Pearson correlation coefficient (R) was calculated. Differences in the volume of the tonsils between smokers and non-smokers (patients who had never smoked or stopped smoking) were evaluated using the Student *t*-test for normally distributed data and the Mann–Whitney U-test for non-normally distributed data.

Furthermore, the difference in V between normal PTs and PTs in patients with PTC was analysed using the paired *t*-test. The difference in vs. between normal PTs in patients with and without PTC was compared using the Mann–Whitney U test. Differences in ΔV% between patients without PTC and those with PTC were compared using the Mann-Whitney U test. A Received Operative Curve (ROC) analysis was performed to evaluate the performance of ΔV% for the identification of patients with and without PTC, and the area under the curve (AUC) was calculated.

The intra-class correlation coefficients (ICCs) were calculated to determine the inter-observer agreement concerning size based on absolute agreement among observers. A *p*-value of <0.05 was considered statistically significant. All statistical analyses used MedCalc^®^ Statistical Software version 22.016 (MedCalc Software Ltd., Ostend, Belgium; https://www.medcalc.org; 2023).

## 3. Results

### 3.1. Patients

Of the 250 patients without PTC, 220 were male and 30 were female, with a median age of 53 years (range from 28 to 78 years). Cigarette consumption was reported for 200/250 patients without PTC. Of the 29 patients with PTC, 21 were male and 8 were female, with a median age of 64 (range of 43–88 years). Of these patients, 13 were staged as T1 disease and 16 as T2 disease.

### 3.2. Sizes of the PTs in Patients Without PTC

The sizes of PTs on each side of the neck and both sides combined, and the ΔV% between the two sides, are shown in Table 1. The volume of the palatine tonsils (PTs) was significantly larger on the right side compared to the left side (*p* < 0.01). The inter-observer agreement for V of PTs showed an ICC of 0.99 (*p* < 0.01).

The median ΔV% for the PTs between the left and right sides was 11.6% (range: 0.1–79.0%) (Table 1). Table 2 shows the numbers of patients with different ΔV% in patients without PTC. Most patients (92.4%) had a ΔV% of ≤30%, and only 4.8% showed a ΔV% of >40% for the PTs (Table 2).

### 3.3. Differences in Size Between Normal PTs and PTs with PTC

The V of the normal PTs and the PTs in patients with PTC are shown in Table 1. Compared with those with PTC, the normal PTs showed a smaller size (*p* < 0.01). Two representative examples of patients without PTC and with ipsilateral PTC are shown in Figure 1. The median ΔV% in patients with ipsilateral PTC was 132.9%, with a wide range of 8.5% to 863.2%. Table 2 shows the numbers of patients with different ΔV% values in patients with PTC. The majority of the patients (89.7%) had a ΔV% of >40%, and only 2/29 (6.9%) patients had a ΔV% of ≤10%. Compared with patients without PTC, patients with PTC showed a greater ΔV% (*p* < 0.01). The use of ΔV% for the identification of patients with and without PTC showed an AUC of 0.95 (95% CI: 0.86–0.99), and the optimal ΔV% threshold of 39.6% achieved a sensitivity of 90.0% (95% CI: 72.6–97.8%), specificity of 95.0% (91.8–97.5%), and accuracy of 94.6% (95% CI: 91.3–97.0%) for the identification of patients with PTC. Compared with normal PTs in patients without PTC, the normal PTs in patients with PTC showed a smaller size (*p* < 0.01).

### 3.4. Association Between the Size of PTs with the Size of Ipsilateral Nodes, Age, and Cigarette Consumption in Patients Without PTC

**Figure 2:**
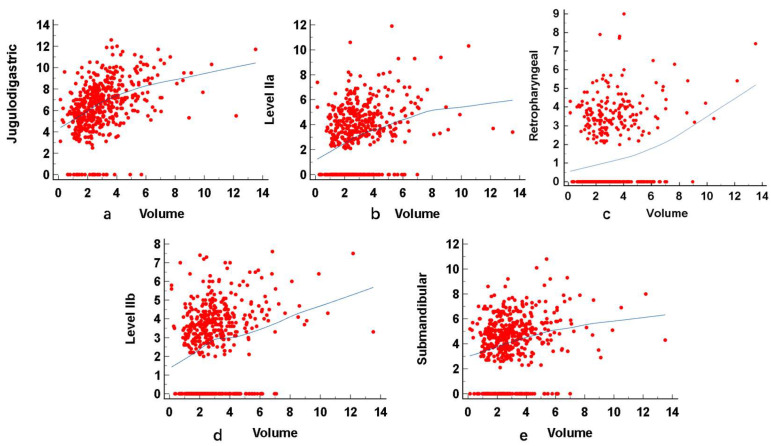
Scatter plots indicating a positive correlation between the volume (cm^3^) of the PTs and the sizes (short axis diameter in mm) of the ipsilateral nodes in the jugulodigastric (**a**), Level IIa (**b**), retropharyngeal (**c**), Level IIb (**d**), and submandibular (**e**). The Pearson correlation coefficients range from 0.15 to 0.46 (all *p* < 0.05).

The sizes (SADs) of the largest node in the ipsilateral neck are shown in Table 3. A positive association was shown between the ipsilateral nodal size in all five upper nodal groups for the PTs (R = 0.15 to 0.46; *p* < 0.05) (Table 4 and Figure 2). A negative association was shown between age and V for the PTs (R = −0.19; *p* < 0.05). Compared with non-smokers, smokers showed no difference in the V of PTs (*p* = 0.07).

## 4. Discussion

Asymmetry in the size of the lymphoid tissues in the right and left PTs is a sign that is used to help detect unknown primary tumours on imaging. However, little attention has been paid to the expected normal asymmetry of the PTs in patients without PTC. In this study, we found that the median discrepancy in the size (ΔV%) of the PT was 12%, and in most (about 95%), the ΔV% was ≤40%. These results suggest that when searching for a primary tumour, PTs with a size discrepancy of >40% should be regarded with the highest level of suspicion.

In order to confirm this finding, we further included patients with early-stage PTC (stage T1 and T2 disease) in the analysis, and the results confirmed that patients with early-stage PTC had a greater ΔV%. The use of the optimal ΔV% of >39.6% achieved a sensitivity of 90% and specificity of 95% for the identification of early-stage PTC. However, smaller discrepancies may also need to be regarded with suspicion, depending on the clinical context. It is worth noting that PET/CT is one of the most advanced imaging modalities that can be applied to detect unknown primary tumour sites. However, studies showed low sensitivity for the identification of primary tumour sites in patients with unknown primary cancers from the head and neck region [19]. Early-stage PTC could be even more difficult to identify on PET/CT, as it may arise from the normal tonsillar tissue. Therefore, the clinical role of PET/CT in the early identification of PET/CT should be further investigated.

In this study, we also found that the normal PT in patients with PTC had a smaller size compared with the normal PT in patients without PTC. This result indicated that patients with ipsilateral PTC may have atrophy of the non-cancerous PT, which also explains the large discrepancy in size between the two PTs; this atrophy might potentially be helpful for the identification of patients with early-stage PTC.

In a minority of patients without cancer, a marked discrepancy in size of up to 78% in the palatine tonsils was observed. The reason for this is unknown, but as normal lymphoid tissues are reportedly symmetrical in children [20,21,22], we suspect that it may be the sequelae of infection or repeated infections that have occurred over time.

In the second part of this study, we evaluated the expected range in size of the PTs and the association with age, smoking, and the size of the nodes that drain these sites. Again, our results showed a wide range between individuals. It is well documented that the lymphoid tissues in the palatine tonsils and nasopharyngeal adenoids decrease in size with age [23,24,25,26,27]. Our results for the PTs agree with the literature, although our results show that it is not unusual for older individuals to have large PTs. Interestingly, smoking did not appear to influence the size of the PTs, suggesting that the PTs may not be the primary lymphoid barrier to smoke.

We have long observed that patients with prominent reactively appearing nodes in the neck often also have prominent lymphoid tissues in the tonsils. Still, to our knowledge, no previous studies have evaluated this potential link. In this study, we confirmed this association, i.e., that a larger nodal size was associated with larger ipsilateral PTs. The size of PTs positively correlated with the size of the nodes in all five nodal groups (retropharyngeal, submandibular, jugulodigastric region, Level IIa (other than jugulodigastric node), and Level IIb nodes). We assume that the stimuli that trigger reactive hyperplasia in the tonsils also trigger a similar response in the lymphoid tissues in the first groups of nodes that drain these sites [28].

There are some limitations in this study. First, although we had a large sample size of adult patients without head and neck cancer for the evaluation of PTs, the evaluation of lingual tonsil was not performed, because the MRIs were initially designated for the evaluation of abnormalities in the upper neck, and so not all of them covered a low enough region to reach the lingual tonsils. Second, other potential infections, such as dental disease and oral infections, were not considered for the analysis, because these data were not retrievable. Third, asymmetry in size in locally advanced PTCs is unknown, as this study did not include this group of patients for analysis. However, the identification of locally advanced PTCs can be easy, as they usually invade adjacent tissues, which are easy to identify on imaging and clinical examinations. Fourth, patients without PTC were initially prospectively recruited for cancer screening programmes using EBV tests and were positive for EBV biomarkers. However, all of the patients from this group were negative for any head and neck cancer on at least the two-year follow-up, and so we believe that the findings from this group of patients are representative. Fifth, the asymmetry in size of the bilateral tonsil for indicating malignancy may not be applied to lymphoproliferative disease in the tonsils, such as lymphoma, as we did not include this group of patients in our analysis. Furthermore, cigarette consumption was irretrievable in 50 patients, so our analysis of the correlation of cigarette consumption could only be performed for 200/250 patients. Finally, when searching for an unknown primary tumour on MRI, small focal areas of signal alternation within the PT or focal bulges in the contour also need to be considered, irrespective of the overall size discrepancy between the two sides.

## 5. Conclusions

The results of this study showed that most adults show a discrepancy in the size of the PTs between the right and left sides. The median discrepancy in size between the PTs was 11.6%, with most (around 95%) showing a difference of ≤40%. An additional analysis that included patients with early-stage PTC (stage T1 and T2 disease) confirmed that patients with early-stage PTC had a greater ΔV% compared with patients without PTC. The use of the optimal ΔV% of >39.6% achieved a sensitivity of 90% and specificity of 95% for the identification of early-stage PTC. Knowledge of the expected range may help select biopsy sites in patients with an unknown primary tumour. However, in a minority of individuals without cancer, a much greater discrepancy in size was observed at both sites. Our results also showed that the normal PT in patients with ipsilateral PTC had a smaller size compared with that in patients without PTC, indicating that atrophy occurred in the normal side of the PTs in patients with ipsilateral PTC, which might be a potential indicator for the identification of PTC.

The size of the PTs correlated positively with the size of the draining nodes, suggesting that the stimuli that trigger reactive hyperplasia of lymphoid tissues in the tonsils also trigger a similar response in the lymphoid tissues in the nodes that drain these sites.

## Figures and Tables

**Table 1:** Volumes of PTs in patients without and with PTCs.

	Overall ^a^	Left ^a^	Right ^a^	Left vs. Right *p*-Value	ΔV% ^b^
Patients without PTCs (cm^3^) (*n* = 250)	3.0 ± 1.7 (0.1–13.5)	2.9 ± 1.7 (0.1–13.5)	3.1 ± 1.7 (0.1–12.2)	**<0.01**	11.6% (0.1–79.0%)
	**Overall ^a^**	**Normal PTs ^a^**	**PTCs ^a^**	**Normal PT vs. PTCs** ***p*-value**	**ΔV% ^b^**
Patients with PTCs (cm^3^) (*n* = 29)	-	1.8 ± 1.2 (0.8–6.9)	5.0 ± 3.4 (1.5–12.9)	**<0.01**	132.9% (8.5–863.2%)

^a^ Data are shown as mean value ± standard deviation (range). ^b^ Data are presented as the median value (range). Bold indicates statistical significance. PT = palatine tonsil; PTC = palatine tonsillar cancer.

**Table 2:** Numbers of patients with volume differences between the two sides of palatine tonsils in patients without and with PTCs.

	≤5%	>5%, ≤10%	>10%, ≤15%	>15%, ≤20%	>20%, ≤25%	>25%, ≤30%	>30%, ≤40%	>40%, ≤50%	>50%
Patients without PTCs	62 (24.8%)	51 (20.4%)	41 (16.4%)	37 (14.8%)	26 (10.4%)	14 (5.6%)	7 (2.8%)	2 (0.8%)	10 (4.0%)
Patients with PTCs	0 (0%)	2 (6.9%)	0 (0%)	0 (0%)	0 (0%)	1 (3.4%)	0 (0%)	2 (6.9%)	24 (82.8%)

Data are shown as the number of patients (percentage).

**Table 3:** The SADs of the largest nodes on the left and right sides in the five upper nodal groups.

		Retropharyngeal ^a^	Submandibular ^a^	Jugulodigastric ^a^	Level IIa ^a^	Level IIb ^a^
SAD (mm)	Right	3.7 ± 1.2 (2.0–9.0)	5.0 ± 1.5 (2.3–8.7)	6.8 ± 1.9 (2.5–11.9)	4.3 ± 1.5 (2.1–11.9)	4.0 ± 1.1 (2.1–7.5)
	Left	3.8 ± 1.1 (2.1–7.9)	4.9 ± 1.3 (2.7–9.3)	6.8 ± 2.0 (3.0–12.6)	4.5 ± 1.5 (2.1–9.4)	3.9 ± 1.1 (2.0–7.6)

SAD: short axis diameter. ^a^ Data are shown as mean value ± standard deviation (range).

**Table 4:** Association between V of PTs and size of upper neck nodes in patients without PTCs.

		Retropharyngeal		Submandibular		Jugulodigastric		Level IIa		Level IIb	
		R	*p*-Value	R	*p*-Value	R	*p*-Value	R	*p*-Value	R	*p*-Value
Palatine Tonsils	Right	0.22	**<0.01**	0.27	**<0.01**	0.34	**<0.01**	0.32	**<0.01**	0.26	**<0.01**
	Left	0.24	**<0.01**	0.15	**0.02**	0.46	**<0.01**	0.29	**<0.01**	0.20	**0.02**

R: Pearson correlation coefficient. Bold indicates statistical significance.

## Data Availability

The datasets generated or analysed during the study could be available once requested to the corresponding authors.

## Abbreviations

EBV	Epstein–Barr Virus
FDG	18F-fluorodeoxyglucose
ICCs	Intra-class correlation coefficients
MRI	Magnetic resonance imaging
PET/CT	Positron emission tomography–computed tomography
PTCs	Palatine tonsillar cancers
PTs	Palatine tonsils
ROI	Region of interest
SAD	Short axial diameter
SCC	Squamous cell carcinoma
